# Carotenoids in Marine Animals 

**DOI:** 10.3390/md9020278

**Published:** 2011-02-22

**Authors:** Takashi Maoka

**Affiliations:** Research Institute for Production Development, 15 Shimogamo-morimoto-cho, Sakyo-ku, Kyoto 606-0805, Japan maoka@mbox.kyoto-inet.or.jp; Tel.: +81-75-781-1107; Fax: +81-75-781-1118

**Keywords:** carotenoids, marine animals, metabolism, food chain, chemosystematic

## Abstract

Marine animals contain various carotenoids that show structural diversity. These marine animals accumulate carotenoids from foods such as algae and other animals and modify them through metabolic reactions. Many of the carotenoids present in marine animals are metabolites of β-carotene, fucoxanthin, peridinin, diatoxanthin, alloxanthin, and astaxanthin, *etc.* Carotenoids found in these animals provide the food chain as well as metabolic pathways. In the present review, I will describe marine animal carotenoids from natural product chemistry, metabolism, food chain, and chemosystematic viewpoints, and also describe new structural carotenoids isolated from marine animals over the last decade.

## 1. Introduction

Since the first structural elucidation of β-carotene by Kuhn and Karrer in 1928-1930, about 750 naturally occurring carotenoids had been reported as of 2004 [1]. Improvements of analytical instruments such as NMR, MS, HPLC, *etc.*, have made it possible to perform the structural elucidation of very minor carotenoids in nature [2,3,4]. 

Marine animals contain various carotenoids that show structural diversity [3,4,5,6,7,8,9]. Among the 750 reported carotenoids found in nature, more than 250 are of marine origin. In particular, allenic carotenoids, except for neoxanthin and its derivatives, and all acetylenic carotenoids originate from marine algae and animals [1].

**Figure 1:**
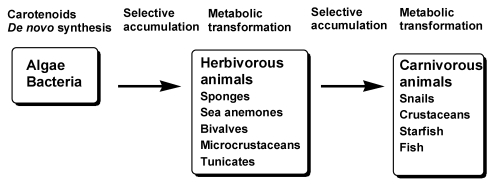
Accumulation and metabolism of carotenoids in marine animals through food chain.

In general, animals do not synthesize carotenoids *de novo*, and so those found in animals are either directly accumulated from food or partly modified through metabolic reactions [5,6,7,8,9], as shown in Figure 1. The major metabolic conversions of carotenoids found in animals are oxidation, reduction, translation of double bonds, oxidative cleavage of double bonds, and cleavage of epoxy bonds. 

Up until 2001, marine animal carotenoids were reviewed by Liaaen-Jensen [5,6], Matsuno [7,8], and Matsuno and Hirao [9]. Since then, there have been no reviews of carotenoids in marine animals. The present review describes progress in the field of carotenoids in marine animals over the last decade.

## 2. Porifera (Marine Sponges)

**Figure 2:**
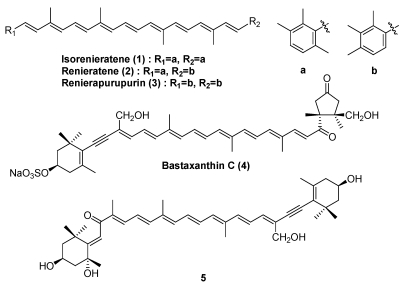
Characteristic carotenoids in marine sponges.

Characteristic carotenoids in marine sponges are shown in Figure 2. Many marine sponges are brilliantly colored due to the presence of carotenoids. Sponges are filter feeders and are frequently associated with symbionts such as microalgae or bacteria [6]. The characteristic carotenoids in sponges are aryl carotenoids such as isorenieratene (**1**), renieratene (**2**), and renierapurpurin (**3**) [6,7]. More than twenty aryl carotenoids have been reported in sponges [1]. Except for sea sponges, aryl carotenoids are found only in green sulfur bacteria [1,6]. Therefore, aryl carotenoids in sponges are assumed to originate from symbiotic bacteria [6,7]. Novel carotenoid sulfates having an acetylenic group, termed bastaxanthins (**4**), were isolated from the sea sponge *Ianthella basta* [1]. Recently, a new acetylenic carotenoid (**5**) was isolated from the marine sponge *Prianos osiros* [10]. Based on the structural similarity, bastaxanthins and compound **5** were assumed to be metabolites of fucoxanthin originating from microalgae.

## 3. Coelenterata (Sea Anemones)

**Figure 3:**
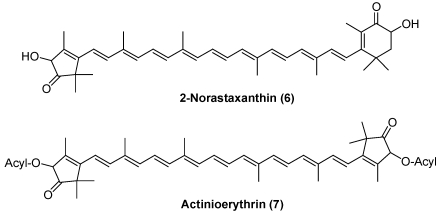
Characteristic carotenoids in sea anemones.

Astaxanthin, which originates from dietary zooplankton, was found in some jelly fish. Peridinin, pyrrhoxanthin, and diadinoxanthin were found in some corals [11]. They originate from symbiotic dinoflagellates. Unique nor carotenoids, 2-nor-astaxnthin (**6**) and actinioerythrin (**7**), have been reported in the sea anemones *Actinia equina* and *Tealia felina* [1] (Figure 3). 

## 4. Mollusca (Mollusks)

Many chitons are herbivorous and feed on attached algae. Major carotenoids found in chitons are lutein, zeaxanthin, fucoxanthin, and their metabolites [12].

Abalone, *Haliotis discus discus*, and turban shell, *Turbo cornutus*, feed on brown and red algae. Carotenoids found in these shells are β-carotene, α-carotene, zeaxanthin, lutein, and fucoxanthin [11].

On the other hand, many sea snails are carnivores. The triton *Charonia sauliae* feeds on starfish. Therefore, astaxanthin (**8**), 7,8-didehydroastaxanthin (**9**), and 7,8,7′,8′-tetradehydroastaxanthin (**10**), characteristic carotenoids found in starfish, were isolated as major carotenoids in triton. Astaxanthin (**8**), originating from dietary microcrustaceans, was found to be a major carotenoid in the whelk *Buccinum bayani*. Alternatively, *Drupella* *fragum* preys upon corals. Thus, peridinin and diadinoxanthin are present as major carotenoids in this sea snail [11]. Carotenoids in sea snails well reflect their diet.

**Figure 4:**
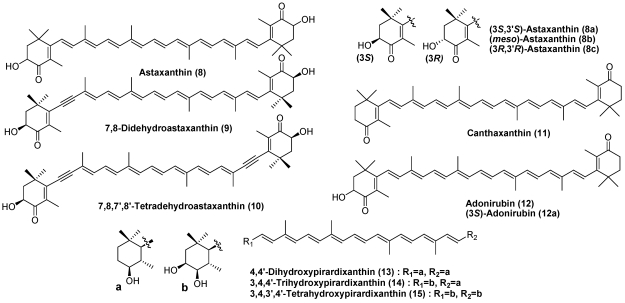
Characteristic carotenoids in sea snails.

Canthaxanthin (**11**), (3 *S*)-adonirubin (**12a**), and (3*S*,3′*S*)-astaxanthin (**8a**) were found to be major carotenoids in the spindle shell *Fushinus perplexus* [13]. Furthermore, a series of carotenoids with a 4-hydroxy-5,6-dihydro-β-end group and/or 3,4-dihydroxy-5,6-dihydro-β-end (**13**-**15**) were isolated from *Fushinus perplexus* [13] (Figure 4). They were assumed to correspond to reduction metabolites of canthaxanthin (**11**), (3*S*)-adonirubin (**12a**), and (3*S*,3′*S*)-astaxanthin (**8a**).

Sea slugs and sea hares also belong to Gastropoda. They are herbivorous and feed on brown and red algae. Several apocarotenoids have been reported in sea slugs and sea hares [1]. A series of 8′-apocarotenal and 8′-apocarotenols derived from β-carotene, lutein, and zeaxanthin were found in the sea hare *Aplysia kurodai* [14]. They are oxidative cleavage products of the polyene chain at C-8 in C_40_ skeletal carotenoids [14].

Bivalves (oyster, clam, scallop, mussel, ark shell, *etc.*) contain various carotenoids that show structural diversity [3,6]. Bivalves accumulate carotenoids obtained from their dietary microalgae and modify them through metabolic reactions. Many of the carotenoids present in bivalves are metabolites of fucoxanthin, diatoxanthin, diadinoxanthin, and alloxanthin [3,6], which originate from microalgae.

**Figure 5:**
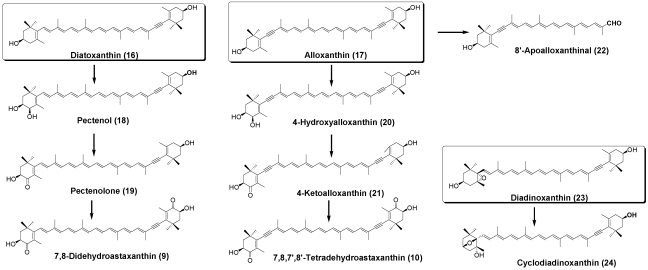
Metabolites of diatoxanthin, alloxanthin, and diadinoxanthin in bivalves.

Oxidative metabolites of diatoxanthin (**16**) and alloxanthin (**17**), such as pectenol (**18**), pectenolone (**19**), 4-hydroxyalloxanthin (**20**), and 4-ketoalloxanthin (**21**), are distributed in scallops and ark shells [3,6,7]. 8′-Apoalloxanthinal (**22**), which is an oxidative cleavage product of alloxanthin, was also found in bivalves [15] (Figure 5).

A novel 3,6-epoxy derivative of diadinoxanthin (**23**), named cycloidadinoxanthin (**24**), was also isolated from the oyster [16] (Figure 5). 

Fucoxanthin (**25**) and its metabolites fucoxanthinol (**26**) and halocynthiaxanthin (**27**) were found to be widely distributed in oysters and clams [3,6,7].

Mytiloxanthin (**28**), which has a unique enol hydroxy group at C-8′ in the polyene chain and a 3′-hydroxy-6′-oxo-κ-end group, is a characteristic carotenoid in marine mussels and oysters [6,7]. Furthermore, three mytiloxanthin analogues containing an allenic end group (**29**), a 3,6-epoxy-end group (**30**), and a 3,4-dihydroxy-β-end group (**31**) were isolated from the oyster [16,17]. Compound **29**, termed allenic mytiloxanthin, was assumed to be a metabolic intermediate from fucoxanthinol to mytiloxanthin. 

Some edible clams have a bright orange or red color due to the presence of carotenoids. Fucoxanthin 3-ester (**32**) and fucoxanthinol 3-ester (**33**) were found to be major carotenoids in *Mactra chinensis* [18], *Ruditapes philippinarum*, and *Meretrix* *petechialis* [19]*.* Amarouciaxanthin A (**34**) and its ester were also identified as major carotenoids in *Paphia amabills* and *Paphia amabillis* [20].

**Figure 6:**
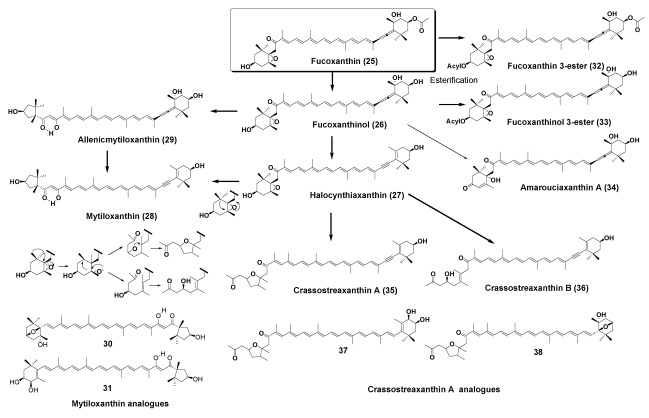
Metabolic pathways of fucoxanthin in bivalves.

Other metabolites of fucoxanthin, crasssostreaxanthin A (**35**) and crassostreaxanthin B (**36**), were isolated from the Japanese oyster *Crassostrea gigas* [21]. Tode *et al.* demonstrated that crassostreaxanthin B could be converted from halocynthiaxanthin by bio-mimetic chemical reactions [22,23]. Further studies of carotenoids in marine animals revealed that crassostreaxanthin A, crassostreaxanthin B, and their 3-acetates were widely distributed in marine bivalves [16,17]. Moreover, two crassostreaxanthin A analogues, **37** and **38**, were isolated from the oyster as minor components [16,17]. Metabolic pathways of fucoxanthin in bivalves are shown in Figure 6.

**Figure 7:**
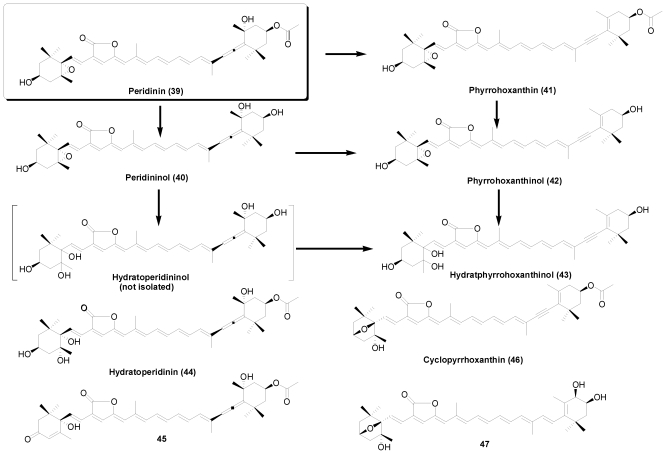
Metabolic pathways of peridinin in bivalves.

Bivalves also feed on dinoflagellates. Peridinin (**39**), a characteristic carotenoid in dinoflagellates with a C_37_-skeletal structure, and its metabolites (**40**-**43**) were also found in some bivalves. Recently, four new C_37_-skeletal carotenoids (**44**-**47**) were isolated from *Crassostrea gigas* [16,17], *Paphia amabillis* [20], and *Corbicula japonica* [24,25]. The metabolic pathways of peridinin in bivalves are shown in Figure 7. As well as fucoxanthin, the major metabolic conversions of peridinin in bivalves are hydrolysis of acetyl group, conversion of the allenic bond to an acetylenic bond, and hydrolysis cleavage of the epoxy ring, as shown in Figure 7. 

**Figure 8:**
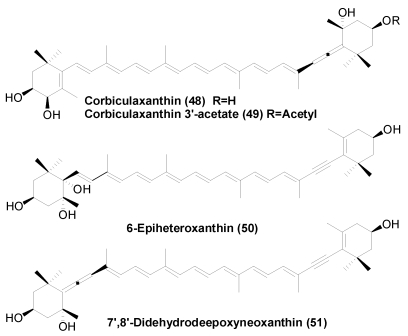
New carotenoids in corbiculaclams.

There are many reports on carotenoids in marine shellfish [6,7]. However, there are few reports on the carotenoids of shellfish inhabiting brackish or fresh water [24,25]. Four new carotenoids, corbiculaxanthin (**48**), corbiculaxanthin 3′-acetate (**49**), 6-epiheteroxanthin (**50**), and 7′,8′-didehydrodeepoxyneoxanthin (**51**), were isolated from the brackish clam *Corbicula* *japonica* and freshwater clam *Corbicula sandai* (Figure 8) [24,25]. 7′,8′-Didehydrodeepoxyneoxanthin (**51**) has an interesting structure, with both allenic and acetylenic moieties. 

Carotenoids found in bivalves provide a key to the food chain as well as metabolic pathways.

Astaxanthin and its esters were found to be major carotenoids in species of octopus and cuttlefish. Their astaxanthins consisted of three optical isomers and originated from dietary zooplankton [26].

## 5. Arthropoda (Crustaceans)

**Figure 9:**
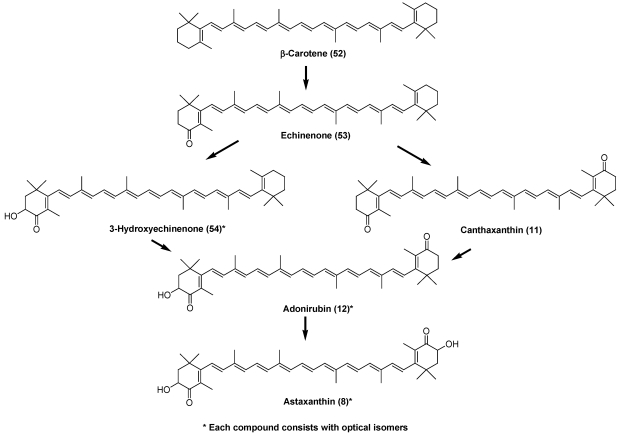
Metabolism of β-carotene in crustaceans.

Carotenoids in the carapace of crustaceans exist as both free and esterified forms. The principal carotenoid in crustaceans is astaxanthin [6,7]. In crustaceans, astaxanthin exists as carotenoproteins such as crustacyanin, and exhibits purple, blue, and yellow colors. Many crustaceans can synthesize astaxanthin (**8**) from β-carotene (**52**), ingested from dietary algae, via echinenone (**53**), 3-hydroxyechinenone (**54**), canthaxanthin (**11**), and adonirubin (**12**), as shown in Figure 9 [6,7]. In many crustaceans, hydroxylation at C-3 (C-3′) in the 4-oxo-β-end group is none-stereo-selective. Therefore, astaxanthin, adonixanthin, and 3-hydroxyechinenone, having a 3-hydroxy-4-oxo-β-end group, present in crustaceans, are comprised of a mixture of optical isomers [6,7]. 

**Figure 10:**
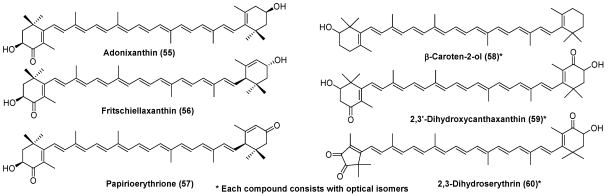
Characteristic carotenoids in crustaceans.

Some crustaceans can convert zeaxanthin to adonixanthin (**55**) and lutein to fritschiellaxanthin (**56**) and papyrioerythrinone (**57**) [1,6,7]. Crustaceans belonging to Isopoda can introduce a hydroxy group at C-2 in the β-end group. This hydroxylation is also none-stereo-selective. Therefore, β-caroten-2-ol (**58**) in the sea louse *Ligia exotica* exists as two optical isomers [1,6,7]. Recently, two new carotenoids, 2,3′-dihydroxycanthaxanthin (**59**) [27] and 2,3-dihydroserythrin (**60**) [28], were isolated from the hermit crab *Paralithodes brevipes* and crawfish *Procambarus clarkii*, respectively (Figure 10).

## 6. Echinodermata (Echinoderms)

Echinenone is a well-known major carotenoid in the gonads of sea urchins and is an oxidative metabolite of β-carotene [6,7]. Echinenone from the gonads of sea urchins was found to have a 9′*Z* configuration (**61**) [29]. 

Starfish are carnivorous and mainly feed on bivalves and small crustaceans. Principal carotenoids in starfish are astaxanthin (**8**), 7,8-didehydroastaxanthin (**9**), and 7,8,7′,8′-didehydroastaxanthin (**10**). They correspond to the oxidative metabolites of β-carotene, diatoxanthin, and alloxanthin, respectively. The crown-of-thorns starfish *Acanthaster planci* is a large, nocturnal sea star that preys upon coral polyps. Recently, four new carotenoids: 4-ketodeepoxyneoxanthin (**62**), 4-keto-4′-hydroxydiatoxanthin (**63**), 3′-epigobiusxanthin (**64**), and 7,8-dihydrodiadinoxanthin (**65**), were isolated from *A.planci* as minor components along with the major carotenoids 7,8-didehydroastaxanthin, peridininol, and astaxanthin, and several other minor carotenoids including 7,8,7′,8′-tetrahydroastaxanthin, diadinoxanthin, diatoxanthin, and alloxanthin [30].

**Figure 11:**
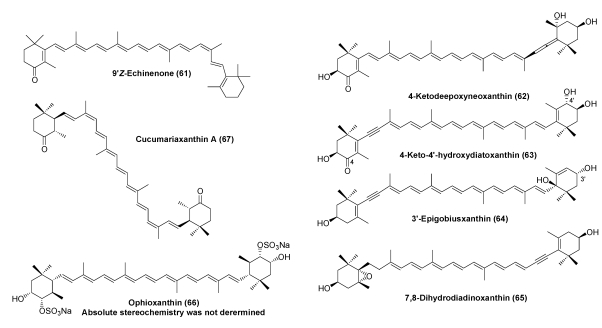
Characteristic carotenoids in echinoderms.

3,4,3′,4′-Tetrahydroxypirardixanthin 4,4′-disulfate, named ophioxanthin (**66**), was reported in the brittle star *Ophioderma longicaudum* [31]. Canthaxanthin and astaxanthin were found in the gonads of sea cucumbers as major components. 5,6,5′,6′-Tetrahydro-β-carotene derivatives with 9*Z*, 9′*Z* configurations, termed cucumariaxanthin (**67**), were isolated from the sea cucumber *Cucumaria japonica* [32] (Figure 11).

Recently, zeaxanthin, astaxanthin, and lutein were identified from spiny sea-star *Marthasterias glacialis* by HPLC-PAD-atmospheric pressure chemical ionization-MS. These carotenoids showed strong cell proliferation inhibition activity against rat basophilic leukemia RBL-2H3 cancer cell line [33]. 

## 7. Protochordata (Tunicates)

As well as bivalves, tunicates are filter feeders. Carotenoids found in tunicates originate from phytoplankton such as diatoms, and are also metabolites of fucoxanthin, diatoxanthin, and alloxanthin [7,8].

**Figure 12:**
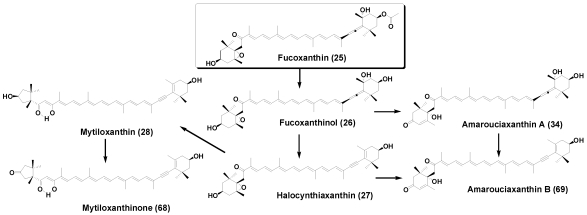
Metabolic pathways of fucoxanthin in tunicates.

Halocynthiaxanthin (**27**), an acetylenic analog of fucoxanthinol (**26**), and mytiloxanthinone (**68**), an oxidative metabolite of mytiloxanthin (**28**), were first isolated from the sea squirt *Halocynthia roretzi* [34]. They are widely distributed in various tunicates. Amarouciaxanthin A (**34**) and amarouciaxanthin B (**69**), having a unique 3-oxo-6-hydroxy-ε-end group, were first isolated from the tunicate *Amaroucium pliciferum* [35] (Figure 12). Peridinin and its metabolites are also found in tunicates.

## 8. Pisces (Fish)

Many fish accumulate carotenoids in their integuments and gonads. On the other hand, Salmonidae fish peculiarly accumulate astaxanthin (**8**) in muscle. Except for catfish, carotenoids in the integuments of fish exist in an esterified form.

**Figure 13:**
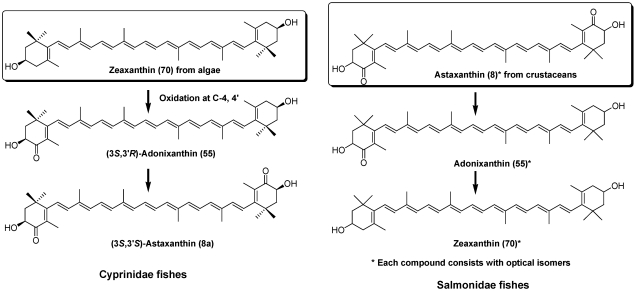
Metabolism of zeaxanthin in Cyprinidae and astaxanthin in Salmonidae fish.

Astaxanthin (**8**) is widely distributed in both marine and fresh water fish. Cyprinidae fish, which inhabit fresh water, can synthesize (3S,3′S)-astaxanthin (**8a**) from zeaxanthin (**70**) by oxidative metabolic conversion (Figure 13). On the other hand, Perciformes and Salmonidae fish cannot synthesize astaxanthin from other carotenoids [6,7,36]. Therefore, astaxanthin present in these fish originates from dietary crustacean zooplankton. Astaxanthin in these marine fish comprises three optical isomers. Perciformes and Salmoidae fish can convert astaxanthin to zeaxanthin [36,37]. Therefore, zeaxanthin in these fish also exists as three optical isomers [38].

**Figure 14:**
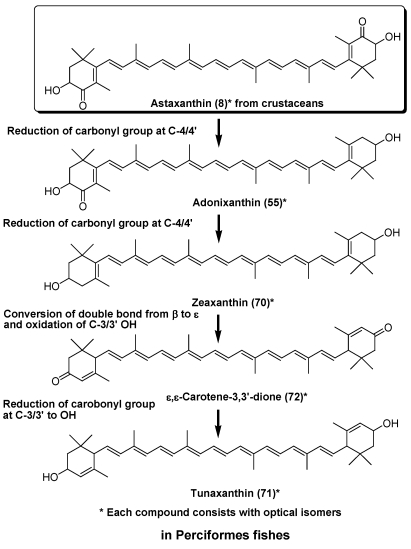
Metabolism of astaxanthin in Perciformes fish.

Tunaxanthin (**71**) is widely distributed in fish belonging to Perciformes. The bright yellow color in the fins and skin of marine fish is due to the presence of tunaxanthin. Feeding experiments involving red sea bream and yellow tail revealed that tunaxanthin (**71**) was metabolized from astaxanthin (**8**) via zeaxanthin, as shown in Figure 14 [7,36]. Carotenoids with a 3-oxo-ε-end group such as ε,ε-carotene-3,3′-dione (**72**) [37] are key intermediates in this metabolic conversion.

Unique apocarotenoids, micropteroxanthins (**73**-**76**), were reported from the integuments of the black bass *Micropterus salmoides* [39]*.* They were assumed to be corresponding oxidative cleavage products of tunaxanthin, lutein, and alloxanthin. 

**Figure 15:**
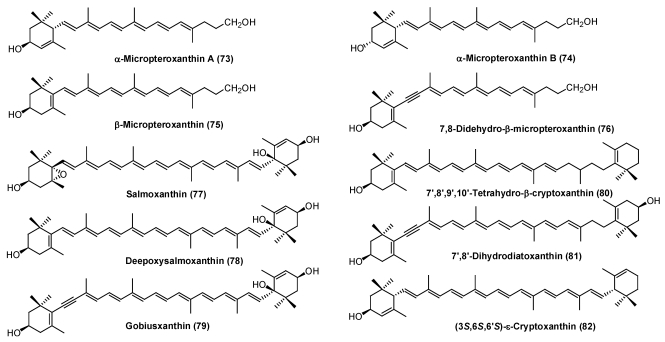
New carotenoids from fish.

Since 2000, there are a few reports on new structures of carotenoids from fish (Figure 15). Carotenoids with a 3,6-dihydroxy-ε-end group, salmoxanthin (**77**), deepoxysalmoxanthin (**78**) (from the salmon *Oncorhynchus keta*) [40], and gobiusxanthin (**79**) (from the freshwater goby *Rhinogobius brunneus*) [41], were isolated. A series of carotenoids with a 7,8-dihydro- and/or 7,8,7′,8′-tetrahydro polyene chain were isolated from the integuments and eggs of the Japanese common catfish *Silurus asotus* [42]. Recently, new carotenoids, 7′,8′,9′,10′-tetrahydro-β-cryptoxanthin (**80**), 7′,8′-dihydrodiatoxanthin (**81**), and (3*S*,6*S*,6′*S*)-ε-cryptoxanthin (**82**), were isolated from the integuments and gonads of the Japanese common catfish as minor carotenoids [43].

## 9. Mammalia (Mammals)

There are few reports available on carotenoids in marine mammals. Only, β-carotene and lutein were reported from the dolphin [44]. The whale is the biggest marine mammal. Whales feed on krill, which is an important dietary source of astaxanthin for marine animals. Therefore, whales might accumulate astaxanthin in the body. 

**Figure 16:**
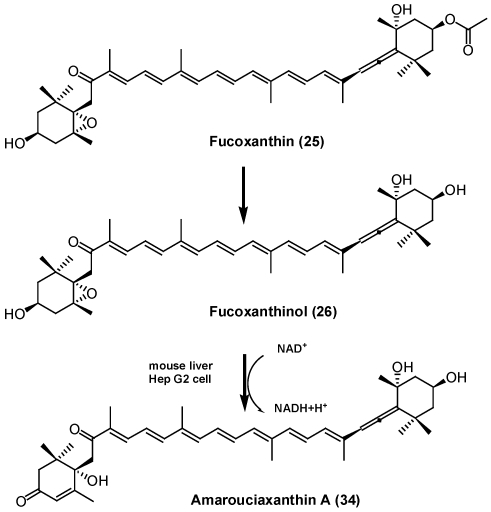
Metabolism of fucoxanthin in mice.

Recently, absorption and metabolism of fucoxanthin (**25**) in mice was investigated. Dietary administrated fucoxanthin was converted to amarouciaxanthin A (**34**) via fucoxanthinol (**26**) in mice [45,46] (Figure 16). This metabolic conversion was also observed in human hepatoma cell HepG2 and required NAD(P)^+^ as a cofactor [45].

## 10. Role of Carotenoids in Marine Animals and Utilization of Carotenoids for Aquaculture

Carotenoids are not essential in the nutritional sense. However, they are beneficial for animal health. It is well-known that carotenoids have an unsubstituted β-end group, such as β-carotene, α-carotene, and the β-cryptoxanthin precursor of vitamin A in animals. Furthermore, canthaxanthin was also converted to retinol in Salmoidae fish. 3-Hydroxy carotenoids: lutein, zeaxanthin, and astaxanthin, were also reported to be precursors of 3,4-dehydroretinol (Vitamin A2) in some freshwater fish [36,47].

Many marine animals accumulate carotenoids in their integuments. Integumentary carotenoids may contribute to photoprotection, camouflage, and signaling such as breeding color. 

Carotenoids have excellent antioxidative activities for quenching singlet oxygen and inhibiting lipid peroxidation. Astaxanthin supplementation in Salmonidae fish suppressed oxidative stress [48,49]. 

Marine animals also accumulate carotenoids in their gonads. Carotenoids are assumed to be essential for reproduction in marine animals. Astaxanthin supplementation in cultured salmon and red sea bream increased ovary development, fertilization, hatching, and larval growth [50]. In the case of the sea urchin, supplementation with β-carotene, which was metabolized to echinenone, also increased reproduction and the survival of larvae [51]. Carotenoids also enhance immune activity in marine animals [52,53]. 

Carotenoids are used for pigmentation in several aquaculture fish. Synthetic and natural astaxanthin from *Phaffia* yeast and *Haematococcus* algae is widely used for the pigmentation of salmon, trout, and red sea bream. Lutein from marigold is also used as a yellow coloration for cultured marine fish such as yellow tail and red sea bream. Zeaxanthin from spirulina is used as a red coloration for goldfish and ornamental carp. 

## 11. Conclusions

In the present review, I have described marine animal carotenoids from natural product chemistry, metabolism, food chain, and chemosystematic viewpoints and also describe new structural carotenoids isolated from marine animals during the last decade. 

In plants and photosynthetic bacteria, biosynthetic roots of carotenoids were identified at the enzymatic and gene level. On the other hand, neither enzymes nor genes for the metabolism of carotenoids in animals have been clarified. Therefore, chemical, biochemical, and analytical approaches are still important to clarify carotenoids in animals.

Interesting new structural carotenoids can still be found in marine animals. The structures of these new carotenoids provide information on the function and food chain, as well as metabolic pathways in marine animals.
